# Living in public rental housing is healthier than private rental housing a 9-year cohort study from Japan Gerontological Evaluation Study

**DOI:** 10.1038/s41598-024-58244-y

**Published:** 2024-03-30

**Authors:** Chie Koga, Tami Saito, Masamichi Hanazato, Naoki Kondo, Masashige Saito, Toshiyuki Ojima, Katsunori Kondo

**Affiliations:** 1 Research Center for Advanced Science and Technology, The University of Tokyo, 4-6-1 Komaba, Meguro-ku, Tokyo, 153-8904 Japan; 2 Center for Gerontology and Social Science, National Center for Geriatrics and Gerontology, 7-430 Morikoka-cho, Obu-shi, Aichi, 474-8511 Japan; 3 Center for Preventive Medical Sciences, Chiba University, 1-33 Yayoi-cho, Inage-ku, Chiba-shi, Chiba 263-8522 Japan; 4 Department of Social Epidemiology, Graduate School of Medicine and School of Public Health, Kyoto University, Kyoto, 606-8501 Japan; 5 Faculty of Social Welfare, Nihon Fukushi University, Aichi, 470-3295 Japan; 6 Department of Community Health and Preventive Medicine, Hamamatsu University School of Medicine, 1-20-1 Handayama, Higashi-ku, Hamamatsu-shi, Shizuoka 431-3192 Japan

**Keywords:** Housing type, Mortality, Environmental factors, Neighborhood unit theory, Older people, Japan, Health policy, Public health

## Abstract

Housing tenure is an important aspect to determine health. However, even though renters tend to have more socioeconomic disadvantages than homeowners, mortality risk between private and public renters compared with homeowners remains unclear. Japanese public rented housing, such as the Urban Renaissance Agency, has been developed for supplying an adequate living environment since 1950s. This study aimed to examine the mortality risk among older Japanese residents living in private and public rented houses compared with those living in owner-occupied houses using 9-year follow-up data. This study drew upon a 9-year follow-up of participants in the Japan Gerontological Evaluation Study, a population-based cohort study of Japanese independent adults aged ≥ 65 years. Mortality from 2010 to 2019 was analyzed for 44,007 respondents. Housing tenure was defined by a questionnaire. Cox regression models were used for calculating the hazard ratio for mortality. Bonferroni correction was used to account for multiple testing between rental houses. Overall, 10,638 deaths occurred during the follow-up period. Compared with housing owners, all rental housing groups had a significantly higher risk of mortality. Among renters, participants who lived in public rental housing had the lowest risk of mortality even after adjusting for sociodemographic characteristics, health status, social status, and environmental status. Multiple testing among renters with Bonferroni correction showed that public renters had 0.80 times (95% CI 0.72–0.89) lower mortality risk than private renters. Although Japanese older adults living in public rental housing had a higher mortality risk than homeowners, this risk was lower than that among private renters. A positive neighborhood environment based on well-planned urban development may have contributed to this result. The results suggest that planned urban development lowers the risk of mortality in older renters in Japan.

## Introduction

Population aging has been considered a challenging issue worldwide. Approximately 30% of Japan’s population are aged ≥ 60 years, which is the highest number globally^1^. Additionally, older adults in Japan are known for their long lifespans^2^. One of the eight age-friendly city topics is housing, which is essential for safety and well-being^3^. A previous study reported that preschool children and retired people spend around 90% of their time at home^4^. Several studies have focused on housing tenure and health from different aspects. For instance, the census longitudinal study conducted by Filakti and Fox^5^ noted that housing tenure is associated with morbidity and mortality. Furthermore, Ellaway and Macintyre^6^ revealed that housing stressors were associated with anxiety and depression. In a study by Macintyre et al.^7^ on 2867 adults in the UK, it was found that the characteristics of the dwelling and its surroundings may help explain the association between housing tenure and health. Another study by Park et al.^8^ using country-level panel data showed that housing cost burden can be associated with population health. Moreover, Park et al.^9^ also investigated 881,220 older adults using population-based linked dataset and revealed that housing assets and income were associated with mortality. Furthermore, Laaksonen et al.^10^ found that subsidized renters had a higher risk of mortality than private renters and owner-occupiers. A recent study from Graetz et al.^11^ using longitudinal data represented that high costs of rent and evictions were associated with mortality. Therefore, a familiar environment, such as housing, can be considered an important health factor in older adults. However, some of those studies did not include factors that contribute to health, such as educational history, work status, and social activities, in their analysis, which may require further analysis. Further research is needed to determine the definition of the housing indicator and the mechanism for preventing adverse health effects.

Although housing is often described as a proxy measure of socioeconomic status (SES), it may also affect health independent of SES. For example, in a study by Macintyre et al.^12^ on 6500 adults in the UK, it was observed that four material asset indicators, i.e., tenure car access, social class, and income, may affect an individual’s health. A study by Do and Kim^13^ on 17,214 older adults in Korea using 2-year follow-up data revealed that individuals in short-term rental houses showed a higher risk of falls and activity limitation due to fear of falling than homeowners did. Furthermore, Pledger et al.^14^ studied 15,626 older adults in New Zealand using pooled data from 2013 to 2016 and revealed that individuals who were in rental tenure were associated with poor health. A study by Morales and Robert^15^ on 1064 older adults in the United States using longitudinal data from 2015 to 2017 revealed that housing cost burden was associated with activity of daily living (ADL)/instrumental ADL limitation over time. A longitudinal study has also investigated the association between housing cost burden and poor psychological well-being^16^. Dalstra et al.^17^, using the national health survey from 10 European countries, demonstrated that even after adjusting for education and income, health differences according to housing tenure were observed and that the difference varies between countries. However, the factors that cause these differences were not determined. Pledger et al. reported that differences in the housing market and public policy may also have an influence^14^. Additionally, urban planning, such as the environment around houses, could also be an important factor.

Rental housing, such as private and public rental housing, can also have an influence on health, and has a different effect depending on the type of rental housing. For example, a study by Digenis-Bury et al.^18^ on 2919 participants showed that public housing residents are more likely diagnosed with several medical conditions, including hypertension, obesity, current asthma, disability, loss of six or more teeth, and feelings of depression, and have poorer health status than other city residents. Furthermore, Windle et al.^19^ interviewed 423 older individuals and revealed that individuals who lived in public rented properties experienced more health problems than those living in owner-occupied and private rented properties. A report showed that private rental houses have poorer quality than public housing and owner-occupied homes in New Zealand, thus having the possibility of different effects on health and well-being^20,21^. A cross-sectional study by Tomioka et al.^22^ on Japanese adults revealed that private renters had poorer self-rated health than other tenure. According to a Japanese White Paper in 2021 of households headed by a married couple with a person aged ≥ 65 years, 87.4% of Japanese older adults were homeowners, 5.5% lived in public rented houses, and 6.9% lived in private rented houses^23^. Another Japanese study showed that older adults living in rental houses are more socially isolated, specifically among private renters but not public renters^24^. Although renters tend to have more socioeconomic disadvantages than homeowners, mortality risk between public and private renters compared with that among homeowners has not been investigated, and only a few studies have compared the mortality between public and private renters^5,8–10^. Thus, considering that most studies have not clearly distinguished the effects between public and private rental housing, further research is needed.

**Figure 1:**
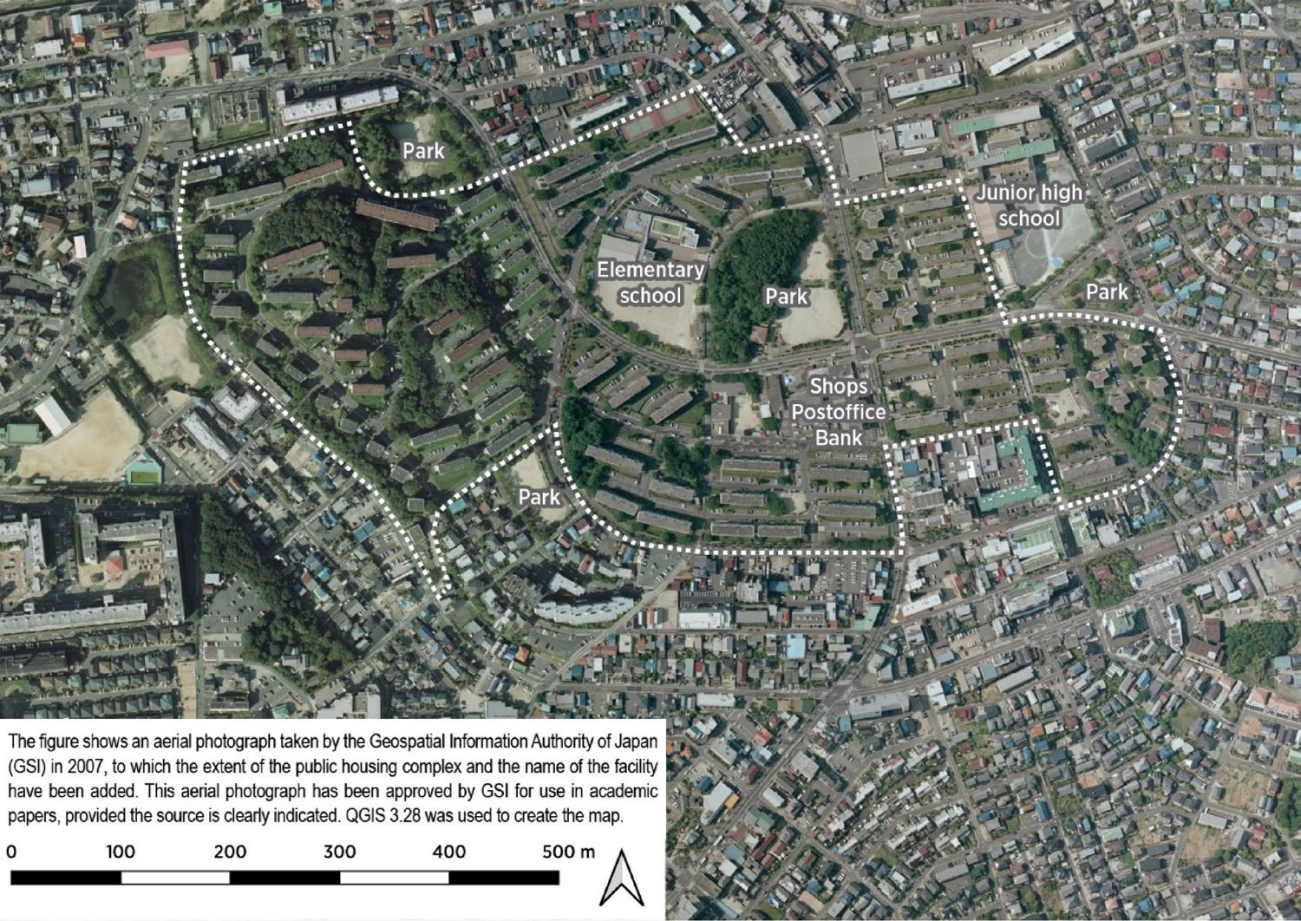
Example of a large public housing complex (including the participants of this analysis): Naruko Housing Complex in Midori Ward, Nagoya City, Aichi Prefecture. The area within the dashed lines is the Naruko Housing Complex. The complex was completed in 1964, with many residential buildings being five-story tall. The main street, which curves to match the terrain, serves as the axis for the area, with a primary school, park, shops, banks, post office, and other facilities located in the center of the complex. The complex also features abundant open spaces with lush greenery between the residential buildings, reflecting the influence of the theory of “the neighboring unit.” During its development, 2196 apartments were built and the planned population was approximately 8000 individuals. Aerial photographs were provided by the Geospatial Information Authority of Japan in 2007.

In Japan, two agencies supply public housing, and differences exist in the backgrounds behind their establishment. One is the local government (municipality or prefecture level). The main purpose of publicly owned housing operated at the municipal or prefectural level is to provide rental housing to low-income individuals who are struggling to find adequate housing. Another is an independent administrative agency, called the Urban Renaissance Agency (UR), which supplies public housing called *Koudan* housing (housing complexes) mainly in urban or suburban areas. The Japan Housing Corporation (JHC), UR’s predecessor organization, was founded in 1955. During the 1950s, 1960s, and 1970s, the JHC built many *Koudan* housing in suburban areas to offset the increasing housing demand during the post-World War II economic boom and later baby boom. The UR is a semi-private, independent administrative agency responsible for housing in Japan other than public housing. As the agency responsible for housing in Japan, it provides housing at market prices, but without the fees (key money or renewal fees) and guarantor requirements common in private rentals in Japan. Public housing developed by the UR refers to the implementation of Perry’s Neighborhood Unit Theory, which involves the deliberate placement of open spaces, commercial facilities, public facilities, and internal streets^25^ (Fig. 1). According to the Ministry of Land, Infrastructure, Transport and Tourism’s 2022 housing economic data, the total number of housing in Japan was 53,616,300, of which 1,922,300 were public rental housing and 747,200 were UR apartments. Furthermore, another report from the Ministry of Land Infrastructure Transport and Tourism showed the distribution of rental housing managed by the UR throughout Japan. According to the report, as of 2012, of all apartment complexes (1732 apartment complexes) managed by the UR, the total for Tokyo, Kanagawa, Chiba, Saitama, and Ibaraki was 911 (52.6%). Furthermore, 124 (7.2%) complexes were located in the Aichi Prefecture; 422 (24.4%) in Osaka, Hyogo, Kyoto, and Nara; 162 (9.4%) in Fukuoka; and 113 (6.5%) in others^26,27^. These data also showed that public housing operated by the UR was concentrated in urban or suburban areas. The average size of UR houses is 46.6–51.9 m^2^^28^. Moreover, as of March 31, 2015, the company announced that it had built 2029 apartment complexes with 883,038 apartments^29^. Based on this data, it has been estimated that each apartment complex has approximately 435 apartments. Several application requirements for UR apartments have been established, such as income requirements and visa (for foreign residents)^30^. The income criterion dictates that the applicant must have an income of four times the rent, thus indicating that not necessarily only low-income families reside in the area^30^.

Public rental housing, such as the UR in Japan, is often larger than private rental housing and is built under planned urban development. If differences in health effects are found between the two, this could clarify some conditions for age-friendly housing. A previous study also revealed that the definition of housing tenure may differ by contextual features, such as historical period, society or region, and culture^7^. Thus, further studies on this topic with different populations and locations are needed. Therefore, this study aimed to examine the risk of mortality among older Japanese residents living in private and public rental houses compared with those living in owner-occupied houses using large-scale 9-year follow-up data. We hypothesized that those who lived in owned houses had the lowest risk of mortality and those who lived in public rental houses had a lower risk of mortality than those who lived in private rental houses. By examining the association between housing tenure and mortality among Japanese older adults, this study can be expected to contribute to creating pieces of evidence for urban planning for healthy older adults.

## Methods

### Study design and participants

**Figure 2:**
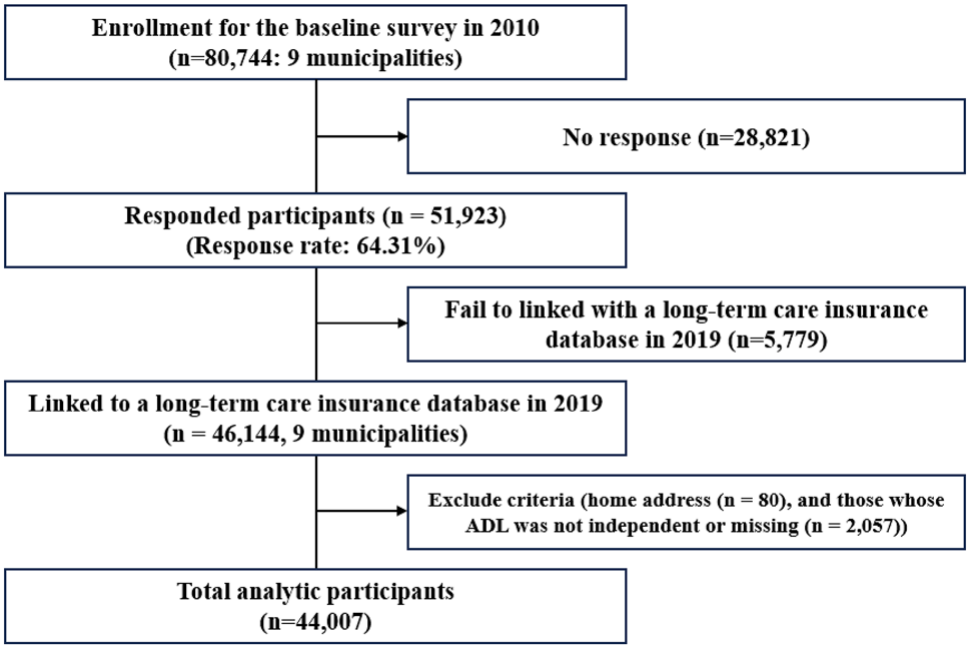
Flow chart of study participants.

The Japan Gerontological Evaluation Study (JAGES) collected baseline data using a mail survey in August 2010 among independent older adults who are ineligible for benefits from the long-term care insurance system in Japan from 11 municipalities^31^. The baseline survey was conducted from August 2010 to December 2011. Self-administered questionnaires were distributed by mail to individuals aged ≥ 65 years who were physically and cognitively independent. The survey was conducted using random sampling in large municipalities and was administered to all eligible residents in small municipalities. Hence, the study participants were independent and relatively healthy older adults. In total, 46,144 have been linked to a long-term care insurance database. These analyses were performed using data from 44,007 participants. We excluded those with missing data, including home address (n = 80), and those whose ADL was not independent or missing (n = 2057). The flow chart of study participants is represented in Fig. 2.

### Mortality outcome

The vital status during the follow-up period from 2010 to 2019 (mean: 3087 days; range: 8–3775 days) was determined by linking data of the self-administered questionnaire to mortality records in the long-term care insurance database. In total, 10,638 deaths occurred in the analytical sample (cumulative mortality = 10,638/44,007; 24.2%). This study examined all-cause mortality instead of cause-specific mortality because death certificate data were unavailable.

### Housing tenure

Housing tenure was defined using a questionnaire. The participants were asked “What type of residence do you live in?” and answers were (1) Owned house; (2) Private rental house; (3) Public rental house; (4) Company housing or dormitories, and (5) Others. Because the participants who answered “living in company housing or dormitories” were few (n = 87), we combined answers (4) and (5) to form the “Others” group. Then, we used these four categories in the analysis.

### Covariates

Based on previous studies on housing status or mortality, we selected demographic factors, health status, social activities, and environmental factors as covariates. For the demographic factors, sex (men or women), age (65–69, 70–74, 75–79, 80–84, or ≥ 85 years), marital status (married, widowed, divorced, unmarried, or others), educational attainment (≤ 9 or ≥ 10 years), equivalent income (low, ≤ ¥1,999,999; middle, ¥2,000,000–3,999,999; or high, ≥ ¥4,000,000), living status (living alone, with family members, or other facilities), longest job held (professional/technical, administrative, clerical, sales/service, skilled/labor, agriculture/forestry/fishery, others, and no occupation), and employment status (worker, retired, or never worked) were selected^6,9,32^. The equivalent income was calculated by dividing the total household income by the square root of the number of household members^33^. Cut-off of the category was followed by a previous report of JAGES^34,35^. For health status, Geriatric Depression Scale (GDS) score, hypertension (yes or no), stroke (yes or no), diabetes mellitus (yes or no), hearing disorder (yes or no), heart disease (yes or no), respiratory disease (yes or no), cancer (yes or no), and body mass index (BMI) were selected^32,36,37^. For social activities, the participation for sports or hobby groups (none of them, one of them, or both of them) and tertile of the total score of social support from family or friends (low, 0–4; middle, 5–7; or high, 8–24) were selected^38^. For social support, four dimensions were used to scale as follows: (1) receiving emotional support, (2) providing emotional support, (3) receiving instrumental support, and (4) giving instrumental support^39^. For the environmental factors, population density as tertile (low, 430–3791 individuals per km^2^; middle, 3818–6549 individuals per km^2^; or high, 6550–27,781 individuals per km^2^) and duration of residence (< 5, 10–19, 20–29, 30–39, 40–49, and > 50 years) were selected^40^.

### Statistical analysis

Descriptive analysis was performed to summarize the characteristics of the participants. Furthermore, owing to the lack of some variables in this analysis, multiple imputations were performed. Twenty multiple imputed datasets, including all measurement variables, were created using the multivariate normal imputation method under a “missing at random” assumption, after which the estimated parameters were combined using Rubin’s combination methods. Cox proportional-hazards model was performed to calculate hazard ratios (HRs) and 95% confidence intervals (CIs) for mortality. We have set three models after the crude. In Model 1, sociodemographic factors (sex, age, marital status, educational attainment, equivalent income, living status, longest job held, and employment status) were added. In Model 2, health status (GDS score, hypertension, stroke, diabetes mellitus, hearing disorder, and BMI) was added. In Model 3, social status (social participation in sports and hobby groups, and social support) was added. In Model 4, environment status (population density and duration of residence) was added. Bonferroni correction was used to account for post hoc multiple testing other than owned house, which is private rental house *versus* public rental house, private rental house *versus* others, and public rental house *versus* others. All statistical analyses were performed using Stata 16/IC (StataCorp, College Station, TX, USA).

### Ethics approval

This study was reviewed and approved by the Ethics Committee of Chiba University (3442) and the Research Ethics Committee involving Human Participants of Nihon Fukushi University (10-05). The study was conducted according to the principles of the Declaration of Helsinki and its later amendments. The JAGES participants were informed that participation in the study was voluntary, and the completion and return of the questionnaire by mail constituted consent to participate in the study. All participants provided written informed consent when they returned a questionnaire.

## Results

Table 1 shows the characteristics of the 44,007 respondents. Of all participants, 37,761 were living in owned houses, 2280 were living in private rental houses, 2490 were living in public rental houses, and 569 were others. Furthermore, the number of participants whose income was low was similar in public rental housing (56.3%) and private rental housing (61.5%), whereas they were higher than participants living in owned houses (38.6%). Moreover, the percentage of participants living alone was also similar in public rental housing (32.1%) and private rental housing (28.6%).

**Table 1:** Baseline characteristics of older Japanese adults according to housing tenure (n = 44,007).

	Total n = 44,007	Owned house n = 37,761	%	Private rental house n = 2280	%	Public rental house n = 2497	%	Others n = 569	%	Missing n = 883	%
Sex											
Male	20,597	17,851	47.3	1077	47.2	1051	42.1	273	48.0	345	39.1
Female	23,410	19,910	52.7	1203	52.8	1446	57.9	313	55.0	538	60.9
Age											
65–69	13,038	11,298	29.9	728	31.9	714	28.6	143	25.1	155	17.6
70–74	13,152	11,194	29.6	743	32.6	801	32.1	194	34.1	220	24.9
75–79	9824	8349	22.1	489	21.4	601	24.1	123	21.6	262	29.7
80–84	5513	4768	12.6	228	10.0	277	11.1	79	13.9	161	18.2
≤ 85	2480	2152	5.7	92	4.0	104	4.2	47	8.3	85	9.6
Marital status											
Married	31,267	28,013	74.2	1092	47.9	1410	56.5	267	46.9	485	54.9
Widowed	9290	7860	20.8	493	21.6	559	22.4	161	28.3	217	24.6
Separated	1576	714	1.9	410	18.0	331	13.3	76	13.4	45	5.1
Unmarried	927	528	1.4	202	8.9	132	5.3	50	8.8	15	1.7
Missing	947	646	1.7	83	3.6	65	2.6	32	5.6	121	13.7
Educational attainment											
> 9	19,549	16,229	43.0	1201	52.7	1337	53.5	247	43.4	535	60.6
≤ 10	23,424	20,766	55.0	999	43.8	1081	43.3	326	57.3	252	28.5
Missing	1034	766	2.0	80	3.5	79	3.2	13	2.3	96	10.9
Income											
Low	18,002	14,589	38.6	1284	56.3	1535	61.5	293	51.5	301	34.1
Middle	14,422	13,205	35.0	490	21.5	493	19.7	115	20.2	119	13.5
High	4079	3898	10.3	63	2.8	47	1.9	44	7.7	27	3.1
Missing	7504	6069	16.1	443	19.4	422	16.9	134	23.6	436	49.4
*Living status*											
Living with someone	35,228	31,577	83.6	1279	56.1	1512	60.6	348	61.2	512	58.0
Living alone	5423	3744	9.9	732	32.1	714	28.6	163	28.6	70	7.9
Missing	3356	2440	6.5	269	11.8	271	10.9	75	13.2	301	34.1
Employment status											
Worker	9028	7800	20.7	577	25.3	426	17.1	86	15.1	91	10.3
Retire	24,843	21,417	56.7	1252	54.9	1584	63.4	288	50.6	276	31.3
Never employed	4877	4312	11.4	191	8.4	228	9.1	58	10.2	82	9.3
Missing	5259	4232	11.2	260	11.4	259	10.4	67	11.8	434	49.2
Longest job											
Professional/technical	6395	5726	15.2	265	11.6	272	10.9	71	12.5	61	6.9
Administrative	2680	2474	6.6	82	3.6	62	2.5	46	8.1	16	1.8
Clerical	6405	5798	15.4	216	9.5	291	11.7	64	11.2	36	4.1
Sales/service	6094	4899	13.0	502	22.0	531	21.3	96	16.9	66	7.5
Skilled/labor	5331	4512	11.9	301	13.2	376	15.1	56	9.8	86	9.7
Agriculture	3013	2869	7.6	36	1.6	45	1.8	14	2.5	49	5.5
Others	5098	4048	10.7	410	18.0	433	17.3	118	20.7	89	10.1
No occupation	2317	2029	5.4	92	4.0	124	5.0	29	5.1	43	4.9
Missing	6674	5406	14.3	376	16.5	363	14.5	92	16.2	437	49.5
GDS											
Normal	26,659	23,659	62.7	1100	48.2	1246	49.9	263	46.2	391	44.3
Mild or severe depression	10,051	8135	21.5	746	32.7	770	30.8	214	37.6	186	21.1
Missing	7297	5967	15.8	434	19.0	481	19.3	109	19.2	306	34.7
Cancer											
No	31,682	27,166	71.9	1576	69.1	1855	74.3	420	73.8	665	75.3
Yes	1958	1672	4.4	105	4.6	115	4.6	35	6.2	31	3.5
Missing	10,367	8923	23.6	599	26.3	527	21.1	131	23.0	187	21.2
Respiratory disease											
No	32,000	27,448	72.7	1593	69.9	1863	74.6	429	75.4	667	75.5
Yes	1640	1390	3.7	88	3.9	107	4.3	26	4.6	29	3.3
Missing	10,367	8923	23.6	599	26.3	527	21.1	131	23.0	187	21.2
Heart disease											
No	28,235	24,258	64.2	1379	60.5	1632	65.4	389	68.4	577	65.3
Yes	5405	4580	12.1	302	13.2	338	13.5	66	11.6	119	13.5
Missing	10,367	8923	23.6	599	26.3	527	21.1	131	23.0	187	21.2
Stroke											
No	33,115	28,379	75.2	1652	72.5	1947	78.0	451	79.3	686	77.7
Yes	525	459	1.2	29	1.3	23	0.9	4	0.7	10	1.1
Missing	10,367	8923	23.6	599	26.3	527	21.1	131	23.0	187	21.2
Diabetes mellitus											
No	28,089	24,138	63.9	1365	59.9	1619	64.8	386	67.8	581	65.8
Yes	5551	4700	12.4	316	13.9	351	14.1	69	12.1	115	13.0
Missing	10,367	8923	23.6	599	26.3	527	21.1	131	23.0	187	21.2
Other diseases											
No	28,716	24,670	65.3	1405	61.6	1638	65.6	382	67.1	621	70.3
Yes	4924	4168	11.0	276	12.1	332	13.3	73	12.8	75	8.5
Missing	10,367	8923	23.6	599	26.3	527	21.1	131	23.0	187	21.2
BMI											
> 18.5	3038	2536	6.7	186	8.2	192	7.7	46	8.1	63	7.1
18.5–24.9	29,946	25,941	68.7	1476	64.7	1625	65.1	332	58.3	524	59.3
25.0–29.9	8473	7257	19.2	446	19.6	497	19.9	90	15.8	164	18.6
≤ 30	2550	2027	5.4	172	7.5	183	7.3	31	5.4	132	14.9
Social participation (sports or hobby group)											
None of them	16,843	13,989	37.0	1114	48.9	1141	45.7	297	52.2	302	34.2
One of them	9227	8278	21.9	330	14.5	398	15.9	117	20.6	104	11.8
Both of them	7676	7185	19.0	158	6.9	225	9.0	35	6.2	73	8.3
Missing	10,261	8309	22.0	678	29.7	733	29.4	137	24.1	404	45.8
Social support											
Low (ref)	15,677	12,789	33.9	1151	50.5	1111	44.5	297	52.2	329	37.3
Middle	12,452	10,972	29.1	479	21.0	690	27.6	148	26.0	163	18.5
High	11,053	10,091	26.7	365	16.0	420	16.8	74	13.0	103	11.7
Missing	4825	3909	10.4	285	12.5	276	11.1	67	11.8	288	32.6
Population density											
Low (ref)	15,258	13,703	36.3	448	19.6	539	21.6	195	34.3	373	42.2
Middle	14,107	12,653	33.5	581	25.5	396	15.9	151	26.5	326	36.9
High	14,642	11,405	30.2	1251	54.9	1562	62.6	240	42.2	184	20.8
Duration of residence, years											
< 5 years (ref)	759	457	1.2	156	6.8	77	3.1	42	7.4	27	3.1
5–9	939	630	1.7	128	5.6	109	4.4	52	9.1	20	2.3
10–19	2130	1632	4.3	205	9.0	186	7.4	59	10.4	48	5.4
20–29	2553	2092	5.5	209	9.2	175	7.0	39	6.9	38	4.3
30–39	4732	4084	10.8	255	11.2	284	11.4	42	7.4	67	7.6
40–49	8037	6799	18.0	453	19.9	543	21.7	97	17.0	145	16.4
> 50 years	24,119	21,526	57.0	823	36.1	1077	43.1	242	42.5	451	51.1
Missing	738	541	1.4	51	2.2	46	1.8	13	2.3	87	9.9

Table 2 presents the HRs with 95% CIs for the association between housing tenure and the risk of mortality. After adjusting for potential confounders in Model 4, participants who lived in private rental houses had 1.45 times (95% CI 1.34–158) higher, those who lived in public rental houses had 1.17 times (95% CI 1.07–1.27) higher, and those who lived in others had 1.21 times (95% CI 1.05–1.40) higher risk of mortality than those who lived in owned houses. The results of post hoc multiple testing among non-home owners indicated a significant difference between private and public renters. Public renters have 0.80 times (95% CI 0.72–0.89) lower risk of mortality than private renters (*p* = 0.0001). The results from other post hoc multiple testing did not indicate significant differences.

**Table 2:** Hazard ratios with 95% confidence intervals for the association of mortality with housing tenure among older Japanese adults (n = 44,007).

	*Crude				*Model 1 (Crude + demographic factor)				*Model 2 (Model1 + Health status)				*Model 3 (Model2 + Social status)				*Model 4 (Model3 + population density and duration of residence)			
	HR	95% CI		*p*	HR	95% CI		*p*	HR	95% CI		*p*	HR	95% CI		*p*	HR	95% CI		*p*
Housing tenure																				
Owned house (ref)	1.00				1.00				1.00				1.00				1.00			
Private rental house	1.58	1.47	1.70	< 0.001	1.56	1.44	1.68	< 0.001	1.49	1.38	1.61	< 0.001	1.47	1.35	1.59	< 0.001	1.45	1.34	1.58	< 0.001
Public rental house	1.21	1.12	1.31	< 0.001	1.20	1.11	1.31	< 0.001	1.17	1.08	1.27	< 0.001	1.14	1.05	1.24	< 0.001	1.17	1.07	1.27	< 0.001
Others	1.57	1.36	1.81	< 0.001	1.39	1.20	1.60	< 0.001	1.30	1.12	1.50	< 0.001	1.25	1.09	1.45	0.002	1.21	1.05	1.40	0.009
Sex																				
Male (ref)					1.00				1.00				1.00				1.00			
Female					0.45	0.43	0.47	< 0.001	0.48	0.45	0.50	< 0.001	0.50	0.47	0.52	< 0.001	0.50	0.47	0.52	< 0.001
Age																				
65–69 (ref)					1.00				1.00				1.00				1.00			
70–74					1.45	1.36	1.55	< 0.001	1.43	1.34	1.53	< 0.001	1.45	1.35	1.54	< 0.001	1.47	1.37	1.57	< 0.001
75–79					2.57	2.41	2.74	< 0.001	2.48	2.33	2.65	< 0.001	2.50	2.34	2.66	< 0.001	2.53	2.37	2.70	< 0.001
80–84					4.50	4.20	4.81	< 0.001	4.24	3.96	4.53	< 0.001	4.18	3.91	4.47	< 0.001	4.24	3.96	4.55	< 0.001
85 ≤					8.07	7.48	8.71	< 0.001	7.51	6.95	8.11	< 0.001	7.33	6.78	7.92	< 0.001	7.45	6.89	8.06	< 0.001
Marital status																				
Married (ref)					1.00				1.00				1.00				1.00			
Widowed					1.15	1.09	1.22	< 0.001	1.16	1.10	1.23	< 0.001	1.11	1.05	1.18	< 0.001	1.10	1.04	1.17	< 0.001
Separated					1.29	1.16	1.44	< 0.001	1.28	1.14	1.42	< 0.001	1.20	1.08	1.34	< 0.001	1.17	1.05	1.31	0.005
Unmarried					1.20	1.05	1.38	0.009	1.17	1.02	1.34	0.029	1.08	0.94	1.24	0.259	1.09	0.95	1.25	0.220
Educational attainment																				
> 9 (ref)					1.00				1.00				1.00				1.00			
10 ≤					0.96	0.92	1.01	0.085	0.97	0.93	1.01	0.184	0.99	0.95	1.04	0.730	1.00	0.96	1.05	0.984
Income																				
Low (ref)					1.00				1.00				1.00				1.00			
Middle					0.90	0.86	0.94	< 0.001	0.92	0.88	0.96	0.001	0.93	0.89	0.98	0.008	0.95	0.90	0.99	0.028
High					0.93	0.86	1.00	0.047	0.96	0.89	1.03	0.261	0.98	0.91	1.05	0.520	1.00	0.93	1.07	0.932
Living status																				
Living with someone (ref)					1.00				1.00				1.00				1.00			
Living alone					1.05	0.98	1.12	0.161	1.03	0.96	1.10	0.458	1.03	0.97	1.10	0.349	1.04	0.97	1.11	0.258
Employment status																				
Worker (ref)					1.00				1.00				1.00				1.00			
Retire					1.19	1.12	1.26	< 0.001	1.14	1.08	1.21	0.000	1.16	1.09	1.23	< 0.001	1.15	1.09	1.22	< 0.001
Never employed					1.24	1.14	1.35	< 0.001	1.18	1.09	1.29	0.000	1.17	1.08	1.27	< 0.001	1.16	1.07	1.26	< 0.001
Longest job																				
Professional/technical (ref)					1.00				1.00				1.00				1.00			
Administrative					1.01	0.92	1.10	0.867	1.01	0.93	1.11	0.759	1.03	0.94	1.12	0.540	1.04	0.95	1.13	0.452
Clerical					1.00	0.93	1.09	0.921	0.99	0.91	1.07	0.755	0.99	0.91	1.07	0.767	0.99	0.92	1.07	0.836
Sales/service					1.11	1.03	1.19	0.008	1.09	1.01	1.18	0.019	1.09	1.01	1.18	0.022	1.10	1.02	1.19	0.012
Skilled/labor					1.02	0.95	1.10	0.551	1.02	0.95	1.10	0.635	1.01	0.94	1.09	0.752	1.01	0.94	1.09	0.774
Agriculture					1.00	0.92	1.09	0.996	1.02	0.93	1.11	0.687	1.02	0.93	1.11	0.684	0.99	0.90	1.08	0.786
Others					1.05	0.97	1.14	0.201	1.05	0.97	1.13	0.219	1.03	0.95	1.11	0.442	1.03	0.96	1.12	0.422
No occupation					1.08	1.01	1.17	0.032	1.09	1.01	1.17	0.023	1.09	1.02	1.18	0.016	1.10	1.02	1.18	0.016
GDS																				
Normal (ref)									1.00				1.00				1.00			
Mild or severe depression									1.24	1.19	1.29	< 0.001	1.18	1.13	1.23	< 0.001	1.17	1.13	1.23	< 0.001
Cancer																				
No (ref)									1.00				1.00				1.00			
Yes									1.84	1.72	1.98	< 0.001	1.84	1.71	1.98	< 0.001	1.85	1.72	1.98	< 0.001
Respiratory disease																				
No (ref)									1.00				1.00				1.00			
Yes									1.60	1.48	1.73	< 0.001	1.60	1.48	1.73	1.732	1.60	1.48	1.73	< 0.001
Heart disease																				
No (ref)									1.00				1.00				1.00			
Yes									1.26	1.20	1.32	< 0.001	1.25	1.19	1.32	< 0.001	1.25	1.19	1.31	< 0.001
Stroke																				
No (ref)									1.00				1.00				1.00			
Yes									1.32	1.14	1.51	< 0.001	1.31	1.14	1.51	< 0.001	1.30	1.13	1.50	< 0.001
Diabetes																				
No (ref)									1.00				1.00				1.00			
Yes									1.21	1.14	1.28	< 0.001	1.21	1.15	1.27	< 0.001	1.21	1.15	1.27	< 0.001
Other disease																				
No (ref)									1.00				1.00				1.00			
Yes									1.05	0.99	1.12	0.14	1.05	0.98	1.12	0.143	1.05	0.99	1.12	0.108
BMI																				
> 18.5 (ref)									1.00				1.00				1.00			
18.5–24.9									0.65	0.61	0.69	< 0.001	0.65	0.61	0.70	< 0.001	0.65	0.61	0.69	< 0.001
25.0–29.9									0.58	0.54	0.63	< 0.001	0.59	0.55	0.64	< 0.001	0.59	0.54	0.63	< 0.001
≤ 30									0.71	0.61	0.82	< 0.001	0.70	0.61	0.82	< 0.001	0.70	0.60	0.81	< 0.001
Social participation (sports or hobby group)																				
None of them													1.00				1.00			
One of them													0.85	0.81	0.89	< 0.001	0.85	0.81	0.90	< 0.001
Both of them													0.76	0.72	0.80	< 0.001	0.76	0.72	0.81	< 0.001
Social support																				
Low (ref)													1.00				1.00			
Middle													0.92	0.88	0.96	0.021	0.92	0.88	0.97	0.001
High													0.84	0.79	0.89	< 0.001	0.84	0.79	0.88	< 0.001
Population density																				
Low (ref)																	1.00			
Middle																	0.95	0.91	1.00	0.030
High																	0.89	0.85	0.94	< 0.001
Duration of residence, years																				
< 5 years (ref)																	1.00			
5–9																	0.85	0.71	1.01	0.063
10–19																	0.70	0.60	0.81	< 0.001
20–29																	0.75	0.65	0.87	< 0.001
30–39																	0.70	0.61	0.80	< 0.001
40–49																	0.68	0.59	0.78	< 0.001
> 50 years																	0.69	0.61	0.79	< 0.001

## Discussion

To the best of our knowledge, this was the first study to investigate the association between housing tenure particularly between residents living in private and public rental housing and mortality risk with four different models. We found that the risk of mortality was the lowest among older adults living in owned houses. Furthermore, the mortality risk was lower in those living in public rental houses than in those living in other types of rental houses.

In this study, even after adjusting for SES, older adults living in owned houses had the lowest risk of mortality. One of the reasons could be that SES is not fully adjusted. Educational attainment, income, and employment status were added as SES in this analysis; however, we did not add other attributes that older individuals may have, such as wealth. Another example of a possible effect from not fully adjusted SES could be the ability to maintain the quality of house. For instance, individuals with higher SES can easily maintain room temperature. In their housing and health guidelines, the World Health Organization strongly recommends controlling indoor cold and heat^41^. Room temperature should be well-balanced indoor temperature to protect health during cold seasons in countries with cold seasons, such as Japan. Older adults with high SES possibly have more capacity to maintain room temperature through, for instance, renovations to maintain room temperature, purchasing a heater or air conditioner, and constantly paying utility bills.

Possibility of a lower risk of mortality among participants living in public rental housing compared with private rental housing is a richer neighborhood environment around public housing that can enhance physical activities and social participation^42^. In Japan, a quantitative supply of houses after World War II^28^ enabled a planned design of the surrounding environment around public rental housing, such as parks, sidewalks, and greenery. Having places suitable for exercise in the community has been associated with a lower risk of heart disease in older men^43^. One of the previous studies revealed that people who are living in a social rented dwelling setup were more likely to be exposed to environmental factors that negatively affect their health, such as noise, crime, and vandalism^7^. The study discusses the issue of low availability of health-promoting environments, such as gardens and community amenities. Another study revealed that lower frailty of older adults was also associated with the accessibility of parks and sidewalks^44^. Lack of grocery stores in the neighborhood increases the risk of death, dementia, and need for long-term care and reduces fresh food intake^32,35,45,46^. Moreover, living in a neighborhood with a higher rate of sidewalk installation was associated with a low risk of dementia in urban areas^47^. Another study revealed that living in a higher subjective neighborhood walkability is associated with lower knee and lower back pain^48^. Hence, living in a positive neighborhood environment, which promotes healthy behaviors, may be associated with a lower risk of mortality.

Another possible mechanism is the positive effect from well-planned environments, such as greenspaces. As previously mentioned, Japanese public houses are well-developed such that all necessities, including green areas, are distributed around the residence^28^. A cross-sectional study by Nishigaki et al. on 126,878 older adults in Japan revealed that living in greener neighborhoods was shown to be associated with lower risk of depression in urban areas. This could be due to the fact that green spaces in an artificial environment may enhance their impression according to the Attention Restoration Theory^49^. Furthermore, the effect of greenness supports reducing the heat island phenomenon, mitigating noise, or improving the air quality^50^. The health benefits of green spaces are also summarized in the meta-analysis^51^.

Moreover, the social dimension may also be a key factor, such as social cohesion. In this study, we added social status (i.e., social participation and support) in Model 3, and the HR decreased by 0.02 after adjusting for social status. A study showed that owning a home is seen as having achieved the “American Dream” and may contribute to maintaining a high subjective sense of well-being^52^. Living in a *Koudan* house represented the “Japanese dream” during the rapid economic growth period (during the 1950s and 1970s) in Japan^28^. Because some individuals continue to live in public houses since their working age, they may have stronger social cohesion or networks than those living in other housing tenures. In some studies, it has been reported that social cohesion increases subjective well-being and reduces the risks of all-cause and cause-specific mortality^53,54^. Moreover, Nishina and Oh^55^ studied 897 older adults in Japan using cross-sectional data and showed that older adults who live in public housing have more social life variety than those living in owned houses. While private rental houses in this study may contain various characteristics, such as building age (new to old) and accessibility to necessities for life (located convenient area to inconvenient area), Japanese public rental houses may have homogeneous characteristics, such as location or access to necessities. Hence, stronger social cohesion may be a reason for our results.

Key strengths of this study were its novelty that the association between housing tenure and the risk of mortality was investigated using cohort data and that participants living in private and public rented houses were compared. Several limitations should be mentioned. First, our analysis was limited to all-cause mortality. Future studies should examine cause-specific mortality to investigate the association between housing tenure and mortality in more detail to determine the impact of environment, such as neighborhood walkability and cardiovascular death. Second, we did not account for the quality of houses. The US Department of Housing and Urban Development outlines eight things to maintain a healthy home: Keep it dry, clean, safe, well-ventilated, pest-free, contaminant-free, well-maintained, and thermally controlled^56^. Japanese old houses tend to be ill thermally controlled, and insulation is inadequate even though it has been stated as a strong recommendation in the housing and health guidelines by the World Health Organization^41^. Japanese census reveals that 59.5% of houses in Japan were built before 2000^57^. Third, we have not analyzed the mechanism of the association; therefore, this association must be further examined. Future analysis should be conducted using data designed to elucidate these issues. Fourth, we have not included the designs of rental housing. It can be imagined that some buildings are designed to promote social interaction in both private and public rental housing complexes; however, such considerations were not considered in this analysis. Fifth, it was impossible to distinguish whether public housing is operated by the local government or the UR. However, according to our JAGES 2019 survey data that allowed us to distinguish them, 55.3% of residents lived in buildings operated by the UR, 33.2% did not, and 11.5% of the data were missing. Sixth, selection bias may be present for residents who want to live in houses operated by the UR because the UR provided housing, particularly for low- to middle-income individuals. Therefore, it is possible that the residents of public housing targeted in this study are not necessarily only from low-income households. Despite the aforementioned limitations, this study discussed important perspectives regarding the association between housing tenure and the risk of mortality.

## Conclusion

In this study, we examined the risk of mortality among older Japanese residents living in private and public rented houses compared with that among those living in owner-occupied houses using 9-year follow-up data. We found that the risk of mortality was lower in those living in public rental houses than in those living in private rented houses and other types of rental houses. As aging leads to frailty, the housing environment may directly affect older individuals. Therefore, rental housing may also be important to consider during planned development, including the neighborhood. Investigating factors related to their living conditions among older populations is important to suggest healthy urban development.

## Data Availability

Data is made available for academic purposes upon request. People can request from the following web page: https://www.jages.net/contact/.

## Abbreviations

ADL: Activity of daily living
BMI: Body mass index
CI: Confidence intervals
GDS: Geriatric Depression Scale
HR: Hazard ratios
JAGES: Japan Gerontological Evaluation Study
JHC: Japan Housing Corporation
UR: Urban Renaissance
